# Diabetes management training for female community health volunteers in Western Nepal: an implementation experience

**DOI:** 10.1186/s12889-018-5562-y

**Published:** 2018-05-21

**Authors:** Bishal Gyawali, Shiva Raj Mishra, Dinesh Neupane, Abhinav Vaidya, Annelli Sandbæk, Per Kallestrup

**Affiliations:** 1 Nepal Development Society (NEDS), Bharatpur, Nepal; 2 Department of Public Health, Aarhus University, Aarhus, Denmark; 3 Department of Community Medicine, Kathmandu Medical College, Kathmandu, Nepal

**Keywords:** Diabetes, Female community health workers, Health education, Screening, Nepal

## Abstract

**Background:**

In the backdroup of a rapidly increasing burden of diabetes in Nepal, a community-based diabetes management program is implemented involving female community health volunteers (FCHVs) under the government run FCHVs program. FCHVs received an intensive one-week training workshop on prevention, control and management of diabetes. The training program was implemented and evaluated to enhance diabetes knowledge of FCHVs and matched according to their literacy level.

**Methods:**

A range of teaching methods were applied, including desk review, active participation, lectures, presentations, discussions, role plays, demonstration and field test. Evaluation of the knowledge attained was done by testing before and after the workshop. Major milestones in the development of the training module were presented from desk review and ending in stakeholder’s participation in reviewing and revising the training package. The qualitative interview transcripts of FCHVs were analyzed thematically.

**Results:**

A 5-day training package was developed through a desk review of interventions using community health workers (CHWs) on diabetes management from similar settings. Training module included home-based blood glucose monitoring and home-based health education on life style counselling delivered through a participatory learning approach. There were 20 participants with a mean age of 47 years (SD ± 5.7). The overall assessment of knowledge of diabetes before-after the training, evaluated by the Diabetes Knowledge Questionnaire (DKQ) showed increases in mean score from 40.4% before training to a mean score of 63.3% after training (Paired *t*-test: *t* = − 11.1, *P* < 0.001, and Wilcoxon test for paired samples: z = − 3.930, *P* = 0.0001). Focus group discussions (FGDs) revealed that FCHVs had a favorable perception of the training program’s effectiveness.

**Conclusions:**

If FCHVs are appropriately trained they may be instrumental in providing counseling and screening for diabetes management in their communities.

## Background

Non-communicable diseases (NCDs) kill nearly 40 million lives each year, accounting for 70% of all global deaths, and if unstopped the number of deaths will reach 52 million annually by 2030 [1]. Globally, more than 422 million adults were living with diabetes in 2014, with almost half remaining undiagnosed (47%); this number is projected to almost double by 2030 [2]. Four out of five people with diabetes live in low-and middle-income countries (LMICs), with 5 million diabetes related deaths yearly [2]. Nutrition transition amidst of industrialization and urbanization is driving the diabetes epidemic in many LMICs. There is evidence of increase in per capita consumption of diets with refined grains, fat, sugar and sweetened beverages, and overall lower intake of fruits and vegetables in these countries [3].

Nepal, like many countries in transition, has experienced an increase in the burden of chronic conditions, including type 2 diabetes. A recent analysis showed 8.4% of adults in Nepal had diabetes, with 1% living in rural areas and much higher, up to 19% in urban areas [4]. This is linked to the changes in lifestyle among the Nepalese populations formerly at less risk in a predominantly agriculture based economy. Many people with diabetes remain untreated and the burden of disease has exerted an extra pressure on the already fragile health system. The World Health Organization (WHO) recommends a community-based program for prevention and control of diabetes as the optimal strategy for mitigating the emerging burden [5].

Community Health Worker’s (CHWs) interventions have documented promise in improving health behaviors and outcomes in developed and developing countries, particularly in those who lack access to adequate health care [6]. CHWs are important elements of care provision for a wide range of health-related services to communities, including maternal and child care, immunization, management of childhood illness, family planning services, nutrition health services and infectious disease control. In fact, they work as bridges between their ethnic, cultural, or geographic communities and health care providers to promote health through outreach, community education, and social support. A growing body of evidence has documented the effectiveness of CHWs in providing hypertension and diabetes care and education to members of hard-to-reach and disproportionately affected communities [7, 8]. However, this type of approach has only rarely been applied to developing countries, including Nepal.

In 1988 the government of Nepal introduced a cadre of female health volunteers, the so-called Female Community Health Volunteers (FCHVs), which has been identified as a successful strategy to address health problems at community level [9]. The FCHVs are lay health workers who form the base of the community-based primary health care system in Nepal, and act as key referral links between the Nepalese health service system and community people. They are, in fact, the frontline CHWs of Nepal who understand the community, use their understanding and experience of the community to build trusting relationships with patients, and serve as a link between the health-care system and the community people in order to facilitate access to services that improve health outcomes. Before the introduction of FCHVs in Nepal’s health system, basic maternal and child health care, including family planning services were provided by a group known as community health leaders, which included both male and female volunteers. However, community women felt uncomfortable with male volunteers discussing their pregnancies and sexual and reproductive health issues. Consequently, female volunteers or FCHVs replaced the male volunteers. Professional health workers act as partners, supervisors and evaluators of FCHVs in Nepal. The FCHVs continuously maintain a record of health activities of their community and report this to the professional health worker. The relationship between FCHVs and professional health workers are characterized by support, mutual respect, trust and partnerships. They are respected and supported by the health professionals with whom they collaborate since they are the ones who are playing important roles in areas where there is limited access to professional health care. This has also earlier been recognized by the government of Nepal, which has acknowledged that the FCHVs have contributed significantly to achieve the milestones of the Nepal’s Millennium Development Goals 4 and 5. The involvement of FCHVs in prevention of diabetes and other NCDs have, however, not been explored until now. In a favorable environment supported by evidences and policies, use of such lay health workers may be instrumental in the future to counteract the burden of diabetes.

The 2015–2020 Multi-sectoral Action Plan for NCDs in Nepal has ambitiously been targeted to extend NCD care to rural populations of the country. The efforts have been scarce to deliver affordable diabetes care due to competing priorities of the government in communicable diseases, maternal, newborn and nutritional deficiency management. The 2015 Nepal Health Facility Survey reported that one in five health facilities offer services for diagnosis and management of diabetes. Although public hospitals are the backbone of health service delivery, the survey found a low availability of diabetes care with only 15.1% of public health facilities offering diabetes related services [10]. Amidst of this, there are some independent efforts from the non-profit sector to model community oriented diabetes care. One of them is the Community-Based Management of Diabetes (COBIN-D) implemented by Nepal Development Society (NEDS) in collaboration with the Ministry of Health of Nepal and Center for Global Health, Aarhus University, which aims to evaluate the effectiveness of lay community health workers in the prevention and management of diabetes. This has come in the backdrop of successful community-oriented interventions on maternal, child and newborn health, and others in NCDs such as community-based management of hypertension (COBIN) [11] and Dhulikhel Heart Study [12] that explored the roles of community health workers in addressing these diverse health problems.

In accordance with evidence [13] that have shown an increased prevalence of diabetes and lack of culturally appropriate strategies for diabetes management in the Nepalese context, we trained FCHVs as a means of facilitating the management of diabetes. The purpose of this study is: 1) to describe the features of a training package for FCHVs in diabetes management at community level, and 2) to assess the effects on FCHV’s knowledge and perceptions regarding diabetes management training.

## Methods

### Study setting

This study is a part of the COBIN-D, which is an ongoing 2-arm clustered randomized controlled trial (RCT) in Lekhnath Municipality-a semi-urban area of the Western Region of Nepal. The COBIN-D aims to determine the effect of FCHVs-led family-based health education on type 2 diabetes prevention and management in adults at community level. As a part of the intervention, FCHVs receive training in diabetes management, screening techniques of blood glucose and blood pressure, body mass index (BMI), and blood pressure monitoring as well as referral of those who have abnormal scores to the nearest health facility. This training comprises a major pillar of the intervention alongside home health education. There were 123 FCHVs in the municipality at the time of study. The participants were trained in the Sishuwa Hospital in the same municipality. Altogether 20 participants were selected conveniently and enrolled in the training sessions, who all completed the training. The training was facilitated by one of the researchers (BG), a medical officer from the local hospital and a health staff from a local non-government organization, all of whom had demonstrable level of experience in working with the government-run FCHVs program.

Assessment of participants’ diabetes knowledge was undertaken 1 week before and 1 week after the training. To obtain qualitative feedback of training, we conducted focus groups discussions (FGD) with FCHVs. The training and data collection was conducted between May and June 2017. Ethical approval was obtained from Nepal Health Research Council (NHRC). Written informed consent was obtained from each participant before the study.

### Development and piloting of training package for FCHVs

The diabetes management training program was a five-day course that aimed to provide FCHVs with knowledge and skills to provide health education regarding the prevention and control of diabetes and to screen blood glucose by using glucometers. The training program was based on a facilitator’s manual entitled ‘Community Based Management of Diabetes in Nepal: Female Community Health Volunteer’s Training Facilitator Guide’ developed by some of the study investigators, the design and content of which was informed by the literature [14]. The manual was developed and designed culturally and contextually appropriate with pictographic information. We designed the training package not tightly spaced spanning over 5 days. Most of the mid-level training packages on behavioral interventions are usually of 2 or 3 days in duration. Given the level of literacy they have and nature of intervention, which involves a fair amount of time in blood glucose monitoring and requires high precision in recording and reporting glucose readings, our package allows FCHVs to involve in skill building sessions alongside building understanding on diabetes management. The training was tailored in a local language. During the 5 days of training, several topics related to diabetes management were discussed and more specifically the ones on: definition of type 2 diabetes mellitus and its major risk factors, measurement of blood glucose, counselling of health promotion messages to community people on reducing major risk factors, and maintenance of proper recording and reporting.

The package is structured into five overall units with 10 lesson plans. Table 1 summarizes a list of curriculum topics covered during the 5 days of diabetes training. The first focuses on the theoretical aspects of diabetes, including classification. The second discusses about type 2 diabetes and its major risk factors. The third included anthropometric measurement techniques, including blood glucose measurement, blood pressure measurement, height and weight. The fourth included counseling techniques and health promotion message on major risk factors. The fifth unit included recording and reporting, and selection of households. Each unit is subdivided into a series of 45 mins’ modules delivered over 5 days.

**Table 1:** Summary of Diabetes prevention and management training materials developed for Female Community Health Volunteers, Nepal). Curriculum topics (10 sessions spanned over 5 days with 240 mins of effective session each day)

Units	Lesssons	Topics	Contents	Materials and methods	Duration
Unit 1	Lesson 1:	Introduction of COBIN-D project; type 2 diabetes and the situation in Nepal	COBIN-D project and its objectives; present status of diabetes in Nepal; role of FCHVs in diabetes prevention and control	Materials: FCHV training manual, facilitator guide, marker, white board	120 min
				Methods: PowerPoint presentations, case study	
				Evaluation: Questionaire and interview	
	Lesson 2:	Theoretical aspects of diabetes	Definition; classification; signs and symptoms; complications	Materials: FCHV training manual, facilitator guide, marker, white board	120 min
				Methods: PowerPoint presentations, discussion	
				Evaluation: Questionaire and interview	
Unit 2	Lesson 3:	Diabetes classification and its major risk factors	Modifiable and non-modifiable risk factors; modifiable risk factor types	Materials: FCHV training manual, facilitator guide, newsprint, marker, white board, metacard, types of tobacco; chewing tobacco, cigarette, beetal leaves (paan), different types of glasses used to drink alcohol in daily life, few pieces of apple and banana	60 min
				Methods: Demonstration, presentation, discussion	
				Evaluation: Questionaire and interview	
	Lesson 4:	Measurement of blood glucose, blood pressure, height and weight	Correctly set up the devices, including glucometer, sphygmomanometer, height measuring tape, and weighing machine; correctly measure the blood sugar; blood pressure and height and weight; instrument care	Materials: FCHV manual, facilitator guide, FCHV register, newsprint, marker, white board, a note book to record results, alcohol pad, batteries, glucometer, test strips, lancets, digital weighing scale, sphygmomanometer, non-stretchable tape, weighing machine	180 min
				Methods: Powerpoint presentation, demonstration, practice, role play	
				Evaluation: Obervation	
Unit 3	Lesson 5:	Counseling and health promotion message on major risk factors and medication compliance	Know the counseling techniques; provide health education for reducing modifiable risk factors	Materials: FCHV training manual, facilitator’s guide, flash card, white board, marker, newsprint	120 min
				Methods: Powerpoint presentation, role play	
				Evaluation: Observation	
	Lesson 6:	Practical session for counseling	Know the practical way of counseling techniques; provide health education practically	Materials: FCHV training manual, facilitator’s guide, flash card	180 min
				Methods: Role play	
				Evaluation: Observation	
Unit 4	Lesson 7:	FCHV visits	Preparation for follow up; when to make the first visit; when to follow up; how to visit	Materials: FCHV manual, facilitator’s guide, FCHV register, follow up time schedule, calendar, referral form	120 min
				Methods: Powerpoint presentation, individual practice	
				Evaluation: Questionaire and interview	
	Lesson 8:	Recording and reporting	Know the detailed format of record, report, record correctly after measurement of blood glucose, blood pressure, weight and height	Materials: FCHV training manual, facilitator’s guide, FCHV register, glucometers, sphygmomanometer, weighing machines and height measuring tapes, batteries, record form, pencil, eraser,	180 min
				Methods: Powerpoint presentation, individual practice	
				Evaluation: Questionaire and interview	
Unit 5	Lesson 9:	Selection of households	Make a list of households that FCHVs have to visit	Materials: FCHV training manual, facilitator’s guide, pen, pencil, list of ward wise respondents with address and contact number	60 min
				Methods: Group discussion	
				Evaluation: Questionaire and interview	
	Lesson 10:	Evaluation and Certification	Visit respondents’ households, measure blood glucose, blood pressure, height and weight, provide counseling on reducing risk factors and record and maintain the FCHV register	Materials: FCHV training manual, facilitator’s guide, FCHV register, notebook, ballpoint pen, name tag, pencil, sharpener, eraser, shoulder bag, feedback form, sphymomanometers, glucometers, non-stretchable tapes and weighing machines	120 min
				Methods:Individual practice Evaluation: Questionaire and interview	

To maintain motivation, a range of teaching methods were applied, including active participation, lectures, presentations, discussions, role plays, demonstration and practice. Questions and comments were welcomed throughout the training with extended time allowed for discussion and sharing of insights by FCHVs, including the facilitators. The program was closed with a remark from participating FCHVs followed by the speech of the local Public Health Officer and the Chairperson of the Health Facility Management Committee (HFMC) of the local hospital. FCHVs received a copy of the manual and a bag containing all relevant handouts, diabetes testing kits, digital sphygmomanometer, and a recording register.

During the household visits, the FCHVs had to screen blood glucose and blood pressure, measure BMI, and deliver the lifestyle counselling intervention, focusing on increasing physical activity, reducing alcohol consumption, avoiding smoking, and nutrition education as well as discussion on diabetes and its complications. In fact, counseling was one of the major training topics that was covered during the training. Volunteers were taught about various counseling techniques with an objective to improve patients’ coping skills, their self-esteem as well as guide them in how to change their risk behavior. For example, during counselling, FCHVs will explain the burden of type 2 diabetes and risk factors in Nepal. These discussions are, in fact, intended to make participants aware of their risk and to increase participants’ acceptance of their diabetes diagnosis that means changing participants’ perceived susceptibility to diabetes. Moreover, if a participant has a high blood glucose, FCHVs will refer them to the nearest health facility and, if on antidiabetic medication, participants will be followed up for adherence to their medication during the visit. Each visit will provide participants with the opportunity to discuss individualized health care needs related to diabetic management with a FCHV as well as discuss goal-setting for health behaviors change using motivational interviewing techniques.

### Quantitative data collection and analysis

Quantitative data collection and analysis was done before and after the training. Participants completed a questionnaire originally designed for use in Nepali to assess the knowledge on diabetes management before and after the completion of the training. The questionnaires were completed anonymously and in strict confidentiality. To assess changes in the participants’ level of diabetes knowledge, we adapted the Diabetes Knowledge Questionnaire (DKQ). The DKQ is a 24 questions tool available and validated in a Nepali version [15]. The DKQ has demonstrated an adequate internal consistency with a reliability coefficient of 0.73. The assumption of linear regression (normality of distribution) was checked using histogram. Incorrect responses to the pre- and post-test were coded as 0, correct answers as 1 and “I don’t know” as 2. Subsequently, it was modified in a dichotomous scale: right answer = 1 and the rest to 0 for wrong answer or unknown response. On the first day, before starting the diabetes training, each FCHV completed the questionnaire as a pre-test. One week after completing the 5 days of diabetes training, all the FCHVs retook the DKQ as a post-test to assess their knowledge. The responses were analyzed using the STATA statistical package (version 13.0). Descriptive statistics were presented as mean scores, standard deviations and percent. The participants’ mean scores before and after the training was compared using a paired sample t-test. The level of significance was set at 0.05.

### Qualitative data collection and analysis

We conducted two focus group discussions (FGDs) among the 20 FCHVs and assessed their personal experience with the training, satisfaction with training and challenges to implementing the training intervention in the community. Two specially trained research assistants with a health professional background who were not the facilitators carried out the FGDs. Qualitative data were analyzed manually using the standard thematic analysis approach [16]. This approach involves getting accustomed to the data through an iterative process of reading the data-set transcripts, generating initial codes, arranging codes into larger categories until generation of a saturated thematic map of the analysis. Following thematic analysis several shared themes emerged from the data. Consensus regarding the nature and coding of emerging themes were reached through regular team meetings during the research process.

## Results

### Quantitative findings

There were 20 participants with a mean age of 47 years (SD ± 5.7). Education levels ranged from 4 to 12 years of schooling with 5% having attained grades 12 years. 85% had worked as FCHVs for more than 10 years 65% were from upper-caste, 60% had up to mid-level education, the mean years of being FCHVs was 18.8 (SD ± 7.3). About 90% were unemployed. Table 2 shows socio-demographic information of participants.

**Table 2:** Socio-demographic information of participants

Variables	M or n	% or SD
Age	47	5.7
Ethnicity		
Upper Caste	13	65
Lower Caste	7	35
Education		
Lower	7	35
Middle	12	60
Upper	1	5
Years of FCHV	18.75	7.3
Employment		
No	18	90
Yes	2	10

*M* Mean, *n* = group size, *SD* = standard deviation

The overall assessment of knowledge of diabetes addressed by the DKQ questionnaire increased from a mean score of 40.4% before training to a mean score of 63.3% after training (Paired *t*-test, *t* = − 11.1, *P* = 0.000 and Wilcoxon test for paired samples: z = − 3.930, *P* = 0.0001). The pre-test mean was 9.7(SD ±1.8) and the post-test mean was 15.2(SD ±2.6). The knowledge status score distribution was approximating a normal distribution. The knowledge distribution of the participants regarding diabetes is shown in Table 3.

**Table 3:** Participants’ knowledge of diabetes before and after training

Items	Pre-test (%)		Post-test (%)		t	*p* value
	True	False	True	False		
Eating too much sugar and other sweet foods is a cause of diabetes.	75	25	90	10	−1.8	0.08
The usual cause of diabetes is lack of effective insulin in the body.	20	80	40	60	−2.1	0.04^a^
Diabetes is caused by failure of the kidneys to keep sugar out of the urine.	15	85	45	55	−2.8	0.01^a^
Kidneys produce insulin.	30	70	45	55	−1.8	0.08
In untreated diabetes, the amount of sugar in the blood usually increases.	65	35	80	20	−1.8	0.08
If I am diabetic, my children have a higher chance of being diabetic.	40	60	60	40	−1.7	0.1
Diabetes can be cured.	85	15	90	10	−0.6	0.5
A fasting blood sugar level of 210 is too high.	40	60	90	10	−3.6	0.001^a^
The best way to check my diabetes is by testing my urine.	25	75	55	45	2.8	0.01^a^
Regular exercise will increase the need for insulin or other diabetic medication.	30	70	45	55	−1.8	0.08
There are two main types of diabetes:	20	80	95	5	7.5	0.000^a^
An insulin reaction is caused by too much food.	5	95	20	80	−1.8	0.08
Medication is more important than diet and exercise to control my diabetes.	25	75	40	60	1.8	0.08
Diabetes often causes poor circulation	70	30	80	20	−1.5	0.16
Cuts and abrasions on diabetes heal more slowly.	70	30	80	20	−3.5	0.002^a^
Diabetics should take extra care when cutting their toenails.	50	50	90	10	2.8	0.01^a^
A person with diabetes should cleanse a cut with iodine and alcohol.	25	75	55	45	−2.1	0.04^a^
The way I prepare my food is as important as the foods I eat.	10	90	30	70	4	0.08
Diabetes can damage my kidneys.	60	40	75	25	1.8	0.08
Diabetes can cause loss of feeling in my hands, fingers and feet.	85	15	95	5	1.4	0.162
Shaking and sweating are signs of high blood sugar.	80	20	95	5	2.9	0.0006^a^
Frequent urination and thirst are signs of low blood sugar.	5	95	45	55	3.5	0.002^a^
Tight elastic hose or socks are not bad for diabetics.	35	65	75	25	−3.5	0.002^a^
A diabetic diet consists mostly of special foods.	15	85	50	50	3.1	0.002^a^

^a^Significant at α = 0.05

### Satisfaction with the training package

All FCHVs attended a follow-up focus group meeting 1 week after the training. FGD revealed that they had favorable perceptions of the training program. A large majority of participants reported that the program met their expectations. For example, the majority mentioned that they were satisfied with the overall training objectives, including its topics and format, facilitators, and the overall administrative arrangements. FCHVs believed that the training was compendious, and this would help to provide diabetes management services, including counseling on diabetes and screening for blood sugar in the future.

‘*The training content was very comprehensive and thorough and the manual was well-organized. The inclusion of pictographs helped me to easily understand things.*’ (FG2)

‘*The course was very useful and effective. I received lots of great information from this very important training. It inspired me on working as an FCHV*.’ (FG2)

‘*The practical demonstrations of checking blood glucose levels and counseling techniques during the training were very relevant to memorizing the lessons learned*.’ (FG2)

### Increased knowledge and skills on diabetes management

A majority of FCHVs reported that the training had raised their level of confidence in providing diabetes management service at community level. They related this with better knowledge of factors that caused diabetes and methods to control diabetes. Moreover, FCHVs demonstrated willingness to share the information learned with their peers.

‘*The training has increased my awareness and confidence in managing diseases. I am now able to diagnose diabetes, categorize diabetes, measure height and weight, calculate body mass index and categorize normal, overweight and obesity status.*’ (FG2)

‘*I used to be very shy, confused, had very low self-confidence in health-related counseling but after the diabetes management training, I can communicate better with patients and families and possibly screen for blood sugar when needed.*’ (FG2)

One FCHV told that she did not know much about diabetes before training by saying: *‘I don’t have any idea about diabetes… I don’t think it can be prevented.’ (FG1),* another participant shared: *‘Had I known about diabetes a year ago or had the training been provided a year ago, perhaps I could have saved my husband from diabetes.’ (FG2).*

Other frequently reported benefits of training included: understanding the importance of treatment adherence and consequences of non-adherence.

‘*Previously, I didn’t have any knowledge about the benefits of continuing on treatment for diabetes managment but now I can advise them about benefits of adhering and not-adhering to medication*’. (FG2)

### Healthcare system-related barriers

FCHVs reported that there were not any governmental health promotion programs to prevent diabetes at community level. One respondent reiterated this by saying:

‘*So far we have not received any such training from the government side*.’ (FG1)

Given the high burden of diabetes in their community, some highlighted that the training on diabetes management should be the responsibility of the government. One respondent highlighted the shortcomings in government training program by saying:

‘*In the situation where we were not yet trained to use a simple thermometer by the government, your program taught us to screen blood glucose, which is far from our imagination …thank you so much for the training!* (FG2)

### Challenges to implementation

Few FCHVs described challenges to delivering the implementation, including lack of monetary incentives and need for refresher course.

‘*We have been given many roles to perform at community level, however, we do not receive any monetary benefit by doing this. Though we are aware that we are volunteers, it is very difficult to work without receiving incentives*.’ (FG2)

‘*Since we are giving service at the grass root level of the community, we need to be up to date with the new information and skill regarding screening, treatment and referral i.e. we need refresher courses timely*’. (FG2)

FCHVs also pleaded to increase the duration of the training. Many felt that it would have been better if the training was longer. One FCHV reported that:

‘*A five-day training is very less for us (to learn). Please provide us for more days*.’ (FG2)

### Suggestions for improvement

FCHVs reported that there is a need for better coordination and collaboration among stakeholders to create a sustainable training program. Such an important training program needs strong collaboration to push/promote the training so that more participants will attend in the future because it is very good for developing countries like Nepal.

Furthermore, all FCHVs found the training very promising. One FCHV shared her feeling as follows:

‘*There are more than 50,000 FCHVs in Nepal, and if all volunteers like us start to measure blood glucose, a lot of people can be screened and treated in early stage*.’ (FG2)

## Discussion

While there is a growing body of evidence on community-based management of NCDs in LMICs, the development side of a training package on the intervention trials is rarely discussed and a gap in the sharing of experiences of lay health workers on NCD management, and more specifically, on diabetes management remains both at local and global level. While evidence on the role of CHWs in addressing NCDs is scarce, available data indicate that CHWs can have a positive impact on the prevention, management, and control of NCDs in LMICs. For instance, a study conducted in Bangladesh, Guatemala, Mexico, and South Africa revealed that CHWs can be adequately trained to effectively screen for, and identify, people at high risk of cardiovascular diseases (CVDs) [17]. Yet another study in Iran documented improved blood pressure and fasting plasma glucose among patients with hypertension and diabetes through CHW-led intervention [18]. Abrahams-Gessel S et al. (2015) [19] conducted a qualitative analysis of in-depth interviews among CHWs, and concluded that CHWs can be an alternative for severe human resource shortages in LMICs by providing training to conduct primary, non-invasive screening for CVD risk in community settings as effectively as the trained health professionals [19]. Based on all these evidences, we summarized the development of a training package for FCHVs on diabetes management, assessed the perceived effectiveness of a training workshop using both qualitative and quantitative evaluation. We saw an increase in proficiency of diabetes knowledge from before to after training (40.4 to 63.3%, respectively); however, this needs some caution for interpretation as it was achieved among only 20 FCHVs. The findings of this study reaffirm those published studies on CHWs diabetes training classes showing a significant improvement in diabetes knowledge following training [20, 21]. Prior education and health literacy is favorable but not necessary for successful training outcomes, as we demonstrated a gain in diabetes knowledge among FCHVs with low levels of education and little to no diabetes literacy.

Our findings provide a convincing example that FCHVs can be trained to serve as health educators in the target community for diabetes management, notwithstanding their current role in maternal, child health and communicable diseases. However, they do raise some important concerns of overload, given the competing roles they now have and no or low incentives for such added components. The overall structure of training package may further assist to improve the package of essential NCD interventions in Nepal and elsewhere, where countries are advancing their CHWs for home-based counseling and glucose monitoring to decrease the increasing burden of diabetes. Educating community people about diabetes, counseling on health promotion message on reducing major risk factors, and screening of blood sugar by trained CHWs is well documented and has shown to be feasible and cost-effective in the management of diabetes [8, 22].

Participants reported deficient knowledge, skills, and abilities in diabetes management before the training program. Similar knowledge gaps have been demonstrated in assessment of CHWs’ knowledge for diabetes management in a previous study [21]. We adopted a relatively simple study tool known as the DKQ to cover major domains for diabetes knowledge such as diet, blood glucose self-monitoring, physical activity and medication intake behavior. Major knowledge gaps were noted among participants in some of the test questions, including ‘Eating too much sugar and other sweet foods is a cause of diabetes’, ‘Usual cause of diabetes is lack of effective insulin in the body’, ‘Diabetes can be cured’, ‘Tight elastic hose or socks are not bad for diabetics’, and ‘Diabetic diet consists mostly of special foods’. The first question of the DKQ: the effect of added sugars on various chronic conditions, including diabetes had a smaller number of correct answers. Our finding is congruent with a previous study conducted in Nepal and elsewhere [23]. However, the question is itself controversial. It must be noted that the root of type 2 diabetes is associated with overall dietary pattern such as meat, dairy products, and fatty foods, aided and abetted by sugary foods and beverages, rather than simply sugar alone. Nonetheless, avoiding added sugars is a helpful step, and it should be taken in addition to increasing the intake of vegetables, fruits, whole grains, and legumes. Our results suggest that the majority of participants in the study, show deficit of knowledge about the causes of the disease which is very relevant aspects towards self-management required for proper control of diabetes. This suggest for a need of educational programs and behavioral change of inappropriate behavior. We believed that conducting a qualitative study would contribute to a deeper understanding of FCHVs needs and experiences. Findings from FGDs demonstrated performing basic tasks, such as anthropometric measurements or categorizing diabetes status can be achieved even among those with no prior education and diabetes literacy. Moreover, our quantitative findings also support these qualitative observations of FCHVs, a positive outcome with the training, including increased knowledge and skills in diabetes management.

While we were hopeful about the benefits of the training program, few were dissatisfied and have concerns as well. Inadequate financial remuneration, including allowances, transportation and training duration and additional burden on FCHVs were the concerns. However, this is not entirely new to our FCHVs, as dissatisfaction with support received, in particular financial compensation, has been a common challenge in other low-income countries [24, 25] as well as in Nepal [26]. An evaluation of CHW programme in Kenya revealed that 88% of the interviewed CHWs acknowledge that reimbursements motivated them to continue serving as CHWs [27]. It has been argued that financial incentives that are too low, irregularly paid or discontinued due to a lack of sustainable programme financing may act as a disincentive to CHWs worse than no payment at all [28]. Indeed, a continued lack of incentives or remuneration over time may become a source of dissatisfaction, lead to high attrition rates, which in turn can have a massive impact on communities and eventually lead to the stoppage of services. The CHWs have a right to fair monetary compensation and asking them to work for free is unjustifiable. This has also been argued by the WHO, which recommends that all trained health workers, including CHWs, should receive adequate wages and/or other appropriate incentives [29, 30]. Payment is, therefore, a necessary component of successful CHW programs. The policy makers need to think towards remunerating CHWs, which will make them appreciated and boost their morale.

While the research indicates promising results, it has a number of limitations. First, the small sample size of FCHVs chosen conveniently could affect the generalizability of our qualitative observations. Secondly, we interviewed those who were willing to take part in our intervention, and very unlikely would there be any complaints from those who voluntarily showed up for participation in the training concerning the training effectiveness. Third, 5 days training, although 2 days longer than the conventional training packages, cannot be claimed to be sufficient, therefore a follow-up evaluation is required to explore the retention and adoption of skills and knowledge in their current portfolio as volunteers. Fourth, the evaluation did not permit to verify the competencies of the trainees. However, despite this, we found the study is unique as it measured the influence of an education intervention on the knowledge of FCHVs.

### Lessons learned

We summarize the lessons in the following points:

- The training provided useful skills that FCHVs may use in interaction with their communities with regard to diabetes management.
- Appropriate incentive scheme should be part of a diabetes management program involving FCHVs.
- A longer duration of training with sufficient opportunities to take part in hands-on skills sessions on glucose monitoring would be useful. This does not necessarily increase the cost of the training program if the whole training is spread over a week without hampering their daily activities.
- Sustainability requires effective collaboration between key stakeholders
- A precaution needs to be taken not to overburden the FCHVs by keeping the package short and comprehensible.

## Conclusions

The development of a culturally and contextually appropriate diabetes management training program resulted in an increase in diabetes knowledge among FCHVs. These findings emphasized the role of the training in improving the knowledge of participants about diabetes management. We noticed a favorable attitude of FCHVs towards diabetes management, coinciding with an increase in knowledge and confidence for diabetes management. These improvements need to be sustained and strengthened. If all CHWs receive such brief training in the management and control of diabetes, major public health benefits are likely to be achieved in many resource-constrained settings with similar networks of CHWs at the primary care level of the health system.

## Abbreviations

CHW: Community Health Workers
COBIN-D: Community Based Management of Diabetes in Nepal
DKQ: Diabetes Knowledge Questionnaires
FCHV: Female Community Health Volunteers
FGD: Focus Group Discussion
HFMC: Health Facility Management Committee
NCD: Non-Communicable Disease
NEDS: Nepal Development Society
NHRC: Nepal Health Research Council
RCT: Randomised Controlled trial
SD: Standard Deviation
WHO: World Health Organization
